# Formation and optimization of three-dimensional organoids generated from urine-derived stem cells for renal function in vitro

**DOI:** 10.1186/s13287-020-01822-4

**Published:** 2020-07-22

**Authors:** Guoliang Sun, Beichen Ding, Meimei Wan, Liang Chen, John Jackson, Anthony Atala

**Affiliations:** 1 Department of Urology, Tongji Hospital, Tongji Medical College of Huazhong University of Science and Technology, 1095 Jiefang Avenue, Qiaokou, Wuhan, 430030 HB China; 2 Department of Urology, First Affiliated Hospital of Harbin Medical University, Harbin, HLJ China; 3 Wake Forest Institute for Regenerative Medicine, Wake Forest University, Winston Salem, NC USA

**Keywords:** Urine-derived stem cells, Organoids, Extracellular matrix, Drug screening, Renal function

## Abstract

**Background:**

Organoids play an important role in basic research, drug screening, and regenerative medicine. Here, we aimed to develop a novel kind of three-dimensional (3D) organoids generated from urine-derived stem cells (USCs) and to explore whether kidney-specific extracellular matrix (kECM) could enable such organoids for renal function in vitro.

**Methods:**

USCs were isolated from human urine samples and cultured with kECM extraction to generate 3D organoids in vitro. Eight densities from 1000 to 8000 cells per organoids were prepared, and both ATP assay and Live/Dead staining were used to determine the optimal USC density in forming organoids and kECM additive concentration. The morphology and histology of as-made organoids were evaluated by hematoxylin and eosin (H.E.) staining, immunofluorescence staining and whole mount staining. Additionally, RT-qPCR was implemented to detect renal-related gene expression. Drug toxicity test was conducted to evaluate the potential application for drug screening. The renal organoids generated from whole adult kidney cells were used as a positive control in multiple assessments.

**Results:**

The optimized cell density to generate ideal USC-derived organoids (USC-organoids) was 5000 cells/well, which was set as applying density in the following experiments. Besides, the optimal concentration of kECM was revealed to be 10%. On this condition, Live/Dead staining showed that USC-organoids were well self-organized without significant cell death. Moreover, H.E. staining showed that compact and viable organoids were generated without obvious necrosis inside organoids, which were very close to renal organoids morphologically. Furthermore, specific proximal tubule marker Aquaporin-1 (AQP1), kidney endocrine product erythropoietin (EPO), kidney glomerular markers Podocin and Synaptopodin were detected positively in USC-organoids with kECM. Nephrotoxicity testing showed that aspirin, penicillin G, and cisplatin could exert drug-induced toxicity on USC-organoids with kECM.

**Conclusions:**

USC-organoids could be developed from USCs via an optimal procedure. Combining culture with kECM, USC-organoid properties including morphology, histology, and specific gene expression were identified to be similar with real renal organoids. Additionally, USC-organoids posed kECM in vitro showed the potential to be a drug screening tool which might take the place of renal organoids to some extent in the future.

## Background

Kidney is considered as an extremely essential and complex organ, which is responsible for several functions including drug metabolism [1, 2]. Because of the ubiquitous drug nephrotoxicity, it is imperative to find appropriate drug screening models [3–5]. Currently, much medical research still relies on traditional two-dimensional (2D) in vitro cell culture or animal models for drug testing and toxicity assays [6]. However, these two models have insurmountable disadvantages. Although 2D cell culture systems are derived from human tissues, their differences from in vivo architecture and physiology inevitably result in the failing in drug clinical transformation [6–9]. They also cannot represent the disease heterogeneity, as they are originally derived from homogeneous cell lines [6]. Otherwise, animal models unavoidably face many problems, such as high cost, ethical issues, and heterogeneity compared to human systems [6].

With the rapid development of regenerative medicine, organoid becomes a valuable research platform for drug development, which has the potential to overcome deficiencies originated from traditional models [1, 6]. Organoids are complex three-dimensional (3D) structures developed from stem cells or organ-specific progenitors through a self-organization process [10]. It is thought to closely recapitulate human in vivo environment because it consists of several cell types and contains multicellular organ structures, displaying architectures and functionalities similar to in vivo organs [11]. So far, organoids of various organs [12–16] have been generated including different renal-like cell types and self-organizing renal organoids [17–23]. They have been verified to be excellent high-throughput drug screening models [6].

Generally, adult somatic stem cells and human pluripotent stem cells (hPSCs) including human embryonic stem cells (hESCs) and human-induced pluripotent stem cells (hiPSCs) are two major sources for organoid generation [24]. However, these approaches face many challenges. Adult somatic stem cells are usually isolated from surgical or puncture specimen, which is invasive and inconvenient [25]. Meanwhile, intrinsic tumorigenicity properties are identified in undifferentiated hiPSCs, and hESCs cannot be applied widely because of medical ethical issues [26]. Thus, we urgently need a simple, safe, and non-invasive method to generate renal organoids.

Urine-derived stem cells (USCs) have been proved to possess the desired regenerative properties, including robust proliferative potential, multipotential differentiation, and paracrine effects [27]. Currently, USCs have been successfully induced to differentiate into urothelium [28]. Moreover, USCs have been applied for personalized modeling of genetic kidney disorders and motor neuron disease [29]. Compared to adult somatic stem cells and hPSCs, USCs can be obtained noninvasively at a less cost and simpler method [27]. This indicates the possibility of organoid generation from USCs and subsequent application in drug screening field.

Renal function including glomerular filtration, renal tubular reabsorption, and endocrine are important evaluation indicators for renal organoids. So far, renal organoids cannot completely recapitulate kidney complex organization, but they enable in vitro modeling of renal function to some extent [30]. Typically, protein Aquaporin-1 (AQP1) which forms a water-specific channel for high permeability to water in kidney proximal tubules and erythropoietin (EPO) which is a hormone mainly secreted by the kidney to stimulate the production of red blood cells are both frequently used as specific markers for renal characteristic and function [31]. Besides, Podocin is a slit diaphragm-associated protein, and Synaptopodin is a cytoskeleton-associated pedicel protein, which both are kidney glomerular markers [32]. Because animal-derived kidney-specific extracellular matrix (kECM) can provide an optimal micro-environment for renal cells, it can affect cell differentiation and contribute much to renal function in 3D culture [30, 33]. O’Neill et al. and Peloso et al. had ever prepared kECM through kidney decellularization and digestion and used it to regulate kidney cell growth and metabolism [34, 35]. Thus, kECM was used in our study as well as AQP1, EPO, Podocin, and Synaptopodin were regarded as specific detection indicators for renal characteristic and function.

Our study aimed to develop USCs with optimal initial density to generate USCs-derived organoids (USC-organoids) capable of renal function. For this purpose, we used the most appropriate concentration of kECM to simulate the in vivo environment. We characterized their morphology, histology, gene expression for kidney-specific markers, compared USC-organoids with renal organoids and finally validated their nephrotoxicity screening ability.

## Methods

### Cell isolation and culture

The USCs were isolated and cultured according to the protocol reported previously [36]. In brief, 14 urine samples (80–400 ml) freshly collected from 3 healthy male individuals were centrifuged at 500*g* for 5 min. After discarding urine supernatant, cell pellets were gently suspended in USC culture medium consisting of embryo fibroblast medium (EFM, Lonza, Basel, Switzerland) and keratinocyte serum-free medium (KSFM, Gibco, Waltham, MA, USA) mixed at a ratio of 1:1 with 10% fetal bovine serum (FBS, Gibco, Waltham, MA, USA). The cells were then cultured in 24-well plates at 37 °C in a 20% O_2_/5% CO_2_ incubator. After 3–5 days, USC individual clones appeared, which was considered as passage 0 (p0). Each clone was trypsinized, and culture medium was changed. When reaching a confluence of 60–70%, they were passaged into 6-well plates (p1). Finally, USCs were expanded in a 150-mm culture dish (p2). USCs at p3–4 were used in all experiments.

Human kidney cells (HKCs) were previously isolated from donor human kidneys not used for transplantation and frozen in liquid nitrogen at passage 0 [37]. They were recovered and cultured in kidney culture medium (KCM) containing equal amount of the following two media: one is KSFM supplemented with 2.5% FBS, 1% penicillin-streptomycin, 0.4% insulin transferrin selenium (Sigma-Aldrich, St. Louis, MO, USA), 0.25% epidermal growth factor and bovine pituitary extract (manufacture’s supplements for KSFM); the other is high glucose Dulbecco’s modified Eagle’s medium (DMEM, Sigma-Aldrich, St. Louis, MO, USA) supplemented with 10% FBS and 1% penicillin-streptomycin. The p1–2 HKCs were applied in the subsequent steps.

### 3D organoids formation

The Gravity PLUS™ 3D culture and assay platform (InSphero, Brunswick, ME, USA) were utilized to generate 3D organoids. During organoid formation, the KCM was applied to culture USCs for renal lineage differentiation. The trypsinized and resuspended USCs in 40 μl KCM were seeded into each well of GravityPLUS plate gently and vertically by 8-channel pipette. All plates were incubated at 37 °C in 20% O_2_/5% CO_2_. Four days later, the 3D organoids were harvested, transferred from GravityPLUS plate into GravityTRAP plate by adding 70 μl KCM into each well with a subtle pipetting pressure. Immediately, the residual medium was aspirated out carefully, and 70 μl fresh KCM was added slowly. For long-term and robust culture, 70 μl KCM per well was refreshed every day. We defined the obtained organoids derived from USCs as USC-organoids.

The HKCs were formed into renal organoids using the same protocol.

### USC-organoids optimization

#### Seeding density optimization by ATP assay

To optimize the initial USC concentration to generate organoids, the individual USCs were calculated and seeded at eight densities from 1000 to 8000 cells (in intervals of 1000 cells) per well respectively in the above procedure. Each 96-well GravityTRAP plate was prepared for each density and incubated for 7 days. Then, the ATP measurement of CellTiter-Glo 3D Cell Viability Assay (Promega, Madison, WI, USA) was utilized to evaluate organoid proliferative ability according to the manufacturer’s instruction. Briefly, 40 μl mixture of CellTiter-Glo 3D reagent and KCM were added into a sample plate and shaken at room temperature (RT) for 30 min. The content in the wells was then moved into assay plate, and their bioluminescence activity was appraised by plate reader. Later, ATP concentration was computed according to the standard curve.

#### kECM concentration optimization by Live/Dead staining

To explore the optimal kECM concentration for USC-organoid viability, we cultured USC-organoids in the mixture of KCM and different ratios of porcine-derived kECM, and then evaluated cytotoxicity by Live/Dead staining.

To prepare for kECM extract, the discarded porcine kidneys in other experiments in our laboratory [31] were obtained and utilized. The production method was modified from previously reported protocol of liver extracellular matrix (ECM) extract [38], which was demonstrated in Additional file 1 (Supplementary Figure 1). Briefly, the kidney decellularization procedure for kECM included complete pre-rinsing through the blood vessels, tissue cutting into blocks followed by slicing into pieces after flash freezing at − 80 °C, reduplicative shaking in distilled water for 3 days, repeated treating with 2% Triton X-100 for 4 days, and finally washing for 2 days for clean removal of Triton X-100. The obtained components were lyophilized and grounded into powder with a freezer mill. ECM powder was mixed with pepsin (Fisher Scientific, Pittsburgh, PA, USA) at a weight ratio of 10:1 and sterilized by gamma irradiation (1 Mrad). Under sterile condition, the mixture was centrifuged at 3000 rpm for 15 min after dissolving in 0.1 mol/L hydrochloric acid. The pellet was discarded repeatedly until the supernatant was clear. The decellularized ECM extracts were further purified by filtering through a 0.2-μm syringe filter (Thermo Fisher Scientific, Rochester, NY, USA) and neutralized by 1 mol/L NaOH to pH 7.0 for ready use.

The USC-organoids at the optimal seeding density (data from section 3.1) were cultured in the KCM supplemented with 5%, 10%, or 15% solubilized kECM extract respectively. Two weeks later, a LIVE/DEAD® Viability/Cytotoxicity Kit (Molecular Probes, Eugene, OR, USA) was implemented to assess the viability of USC-organoids in conditioned medium with the above three kECM concentrations. Simply, the organoid samples were carefully transferred to Eppendorf tubes with top-cut 1 mL pipette tips and incubated with a reagent working solution consisting of 0.5 μL calcein AM and 2 μL Ethidium homodimer-1 in 1 mL KCM for 30–45 min at RT. Washed twice by phosphate-buffered saline (PBS), the samples were observed under an Olympus FV10i confocal microscope. ImageJ was used to quantify the average fluorescence brightness.

## Histology evaluation for 3D organoids

We prepared three groups of samples for the following assessments: USC-organoids (optimized seeding density from the “Seeding density optimization by ATP assay” section), USC-organoids with kECM (optimized seeding density from the section “Seeding density optimization by ATP assay” section and optimized kECM concentration from the “kECM concentration optimization by Live/Dead staining” section), and renal organoids (optimized HKCs seeding density).

### Hematoxylin and eosin staining

On the 7th day of culture, 3D organoid samples were washed triply by PBS for 15 s and fixed with 4% paraformaldehyde for 1 h at RT. After 3 times wash by PBS again, all samples were immersed into preheated and dissolved HistoGel (Thermo Fisher Scientific, Waltham, MA, USA), thrown into 60% EtOH, and treated by dehydration and paraffin embedding. Followed with deparaffinization and hematoxylin and eosin (H.E.) staining process, sample slides were viewed using a Zeiss Axiovert 200M microscope.

### Immunofluorescence staining

The paraffin slides from the “Hematoxylin and eosin (H.E.) staining” section without H.E. staining were chosen for immunofluorescence staining. All slides were incubated at 60 °C for 2 h for deparaffinization, washed by ddH_2_O, and transferred to microwave antigen retrieval in citrate buffer (pH = 6.0) at 95 °C for 10 min. Let the slides cool at RT for 1.5 h and block them with protein blocking buffer (Dako, Carpinteria, CA, USA) for 1 h at RT. After incubation with 1:100 diluted specific rabbit anti-human primary antibodies for AQP1 (#Ab15080, Abcam, Cambridge, MA, USA), EPO (#Ab126876, Abcam), Podocin (H-130, Santa Cruz Biotechnology, Santa Cruz, CA, USA), and Synaptopodin (P-19, Santa Cruz Biotechnology) overnight at 4 °C, corresponding secondary antibodies were carried out to treat washed samples for 1 h in dark at RT. The immunofluorescence signals from paraffin sections were imaged by Zeiss Axiovert 200M microscope. ImageJ was used to measure the average fluorescence brightness.

### Whole mount staining

The 3D organoids were transferred to chamber slides (Thermo Fisher Scientific, Waltham, MA, USA) and washed in PBS repeatedly. Followed with fixation in 4% paraformaldehyde for 1 h, the 3D organoids were permeabilized in 0.2% Triton X-100 for 30 min and washed by PBS again. Sequentially, organoids were incubated in protein blocking buffer for 1 h at RT, in which the primary antibodies for AQP1 and EPO were diluted. On the next day, corresponding secondary antibody was applied. The same primary and secondary antibodies as mentioned above were used except that the dilution of the secondary antibody was 1:500. The fluorescence signals were visualized by Olympus FV10i confocal microscope. ImageJ was used to evaluate the average fluorescence brightness.

## RT-qPCR

Total mRNA was extracted from 3D organoids by a RNeasy Plus Mini Kit (Qiagen, Valencia, CA, USA). The cDNA was synthesized by QuantiTect Rev. Transcription Kit (Qiagen), and the quantitative PCR was then performed by a QuantiTect SYBR Green PCR Kit (Qiagen) using the 7300 Real-Time PCR system (Applied Biosystems, Foster City, CA, USA). The primer sequences were as follows (5′-3′): GAPDH (forward GTCATCATCTCCGCCCCTTCTGC, reverse GATGCCTGCTTCACCACCTTCTTG); AQP1 (forward CTGGGCATCGAGAT CATCGG, reverse ATCCCACAGCCAGTGTAGTCA); EPO (forward GGAGGCCGAGAATATCACGAC, reverse CCCTGCCAGACTTCTACGG); Podocin (forward ACCAAATCCTCCGGCTTAGG, reverse CAACCTTTACGCAGAACCAGA); Synaptopodin (forward CCCAAGGTGACCCCGAAT, reverse CTGCCGCCGCTTCTCA) (Sangon, Shanghai, China).

## Function assay

### Drug toxicity test

The 3D organoids from three groups were treated with three drugs respectively, aspirin, penicillin G, and cisplatin, for nephrotoxicity test. Aspirin, penicillin G, and cisplatin (all from Sigma-Aldrich, St. Louis, MO, USA) were chosen for drug toxicity test because they were proved to be excreted by glomerulus and renal tubules, respectively [39]. The working concentration of these three drugs was all 200 μM, and the drug treatment time was 48 h [39]. Organoids were then homogenized and tested by γ-glutamyltransferase (GGT) Activity Colorimetric Assay Kit (Sigma-Aldrich).

### Dose-response curves and the half maximal inhibitory concentration estimation

We transferred organoid samples into 96-well plates and treated them with cisplatin in DMSO (Sigma-Aldrich) at a gradient concentration of 0.1, 0.2, 0.4, 0.8, 1.6, 3.2, and 6.4 mM. Twenty-four hours later, the relative cell survival was evaluated by MTS Kit (Promega, Madison, WI, USA) according to the manufacturer’s protocol. Relative cell survival was defined as the ratio of the number of viable cells in the drug-containing medium and the drug-free medium. “Relative cell survival” values and drug concentrations were fitted to dose-response curves to estimate the half maximal inhibitory concentration (IC_50_) values.

### Statistical analysis

In ATP assay, six replicated wells for each cell density were examined. In Live/Dead staining, histology evaluation, and RT-qPCR, three samples for each group were evaluated. In GGT activity measurement and drug screening experiment, five and three replicated samples for each group were used respectively.

The data were presented as the mean ± standard deviation (SD). Comparisons between two groups were performed using a two-tailed Student’s *t* test. A value of *P* < 0.05 was considered as statistically significant. *P* < 0.05, *P* < 0.01, and *P* < 0.001 were marked as “*”, “**”, and “***” respectively.

## Results

### Morphology of 2D culture cells

**Fig. 1:**
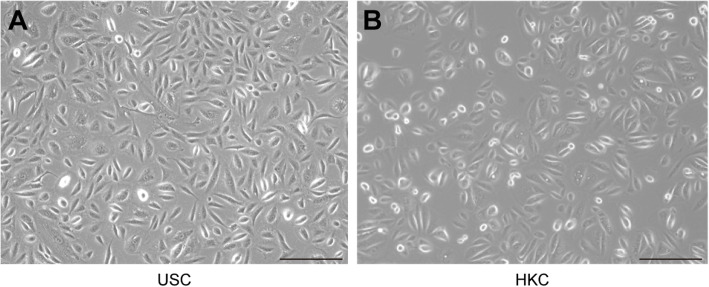
Morphology of USCs and HKCs. **a** The rice grain-like USCs were observed after initial seeding. **b** The HKCs looked more rounded and smaller than USCs in size (scale bar 200 μm)

The morphology of 2D-cultured USCs and HKCs are shown in Fig. 1. Seen from Fig. 1a, the USCs were plump, compact, and bright, showing a rice grain-like appearance after initial seeding. The HKCs looked more rounded than USCs in Fig. 1b. The other properties of USCs have been reported in a previous article [40].

### Morphology and optimization of USC-organoids

#### Morphology observation

**Fig. 2:**
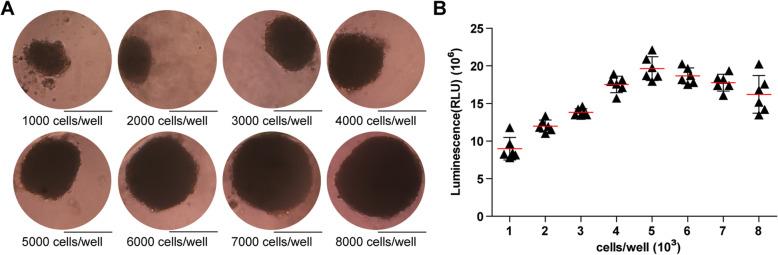
Morphology observation of USC-organoids and cell density optimization via proliferation assay. **a** Morphology of 3D USC-organoids generated from different cell density was observed. The size of the organoids grew larger with the increasing initial cell density (scale bar 200 μm). **b** ATP measurement for proliferation was performed in USC-organoids from different cell density. There was a non-linear relationship between proliferative ability and cell density. The proliferative ability increased with the ascent of initial USC concentration and reached a peak at 5000 cells/well, after which the proliferative ability began to descend. These indicated that suitable size was helpful for the balance of live cells and nutrition supply. Therefore, the 5000 cells/well was the optimal cell density for organoid culture in our study (six replicated wells for each cell density, data presented as mean ± SD)

USCs were seeded at a density of 1000, 2000, 3000, 4000, 5000, 6000, 7000, 8000 cells/well, respectively. Organoids formed at day 3 and were observed at day 7. Under the microscope, USCs were organized into a spheroid in a 3D pattern. Generally, with the USC amount increasing, the organoids became larger and more compact. Theoretically, organoid function will be better and closer to normal tissues if they carry more cells. However, as the volume increases, it is harder for nutrient solution to penetrate inside, so the cells in the sphere center might die due to lack of nutrition. On the basis of our previous results [39], the relatively ideal 3D organoid is about 200–300 μm at diameter, which corresponds to USC-organoids with the seeding density of 3000–6000 cells/well in this experiment. In Fig. 2a, the size of the organoids grew larger ranging from about 160 μm (1000 cells/well) to almost 400 μm (8000 cells/well) with the increasing initial cell density. HKC organoid morphology displayed the same pattern [39].

#### Proliferation assay for cell density optimization

Additionally, ATP activity test was designed to determine USC-organoid proliferative ability. On day 14, these organoids were harvested and the ATP assays were conducted. The results clearly showed that the USC-organoid proliferative ability increased with the ascent of initial USC concentration and reached a peak at 5000 cells/well, after which the proliferative ability began to descend, as shown in Fig. 2b. These results demonstrated that the optimal initial USC concentration for organoid formation was 5000 cells/well, whose corresponding organoid diameter was about 250 μm. Combined with the above morphological results, we concluded that 5000 cells/well was the optimal seeding density for USCs to form organoids and it was applied for further experiments.

#### Viability assay for kECM concentration optimization

**Fig. 3:**
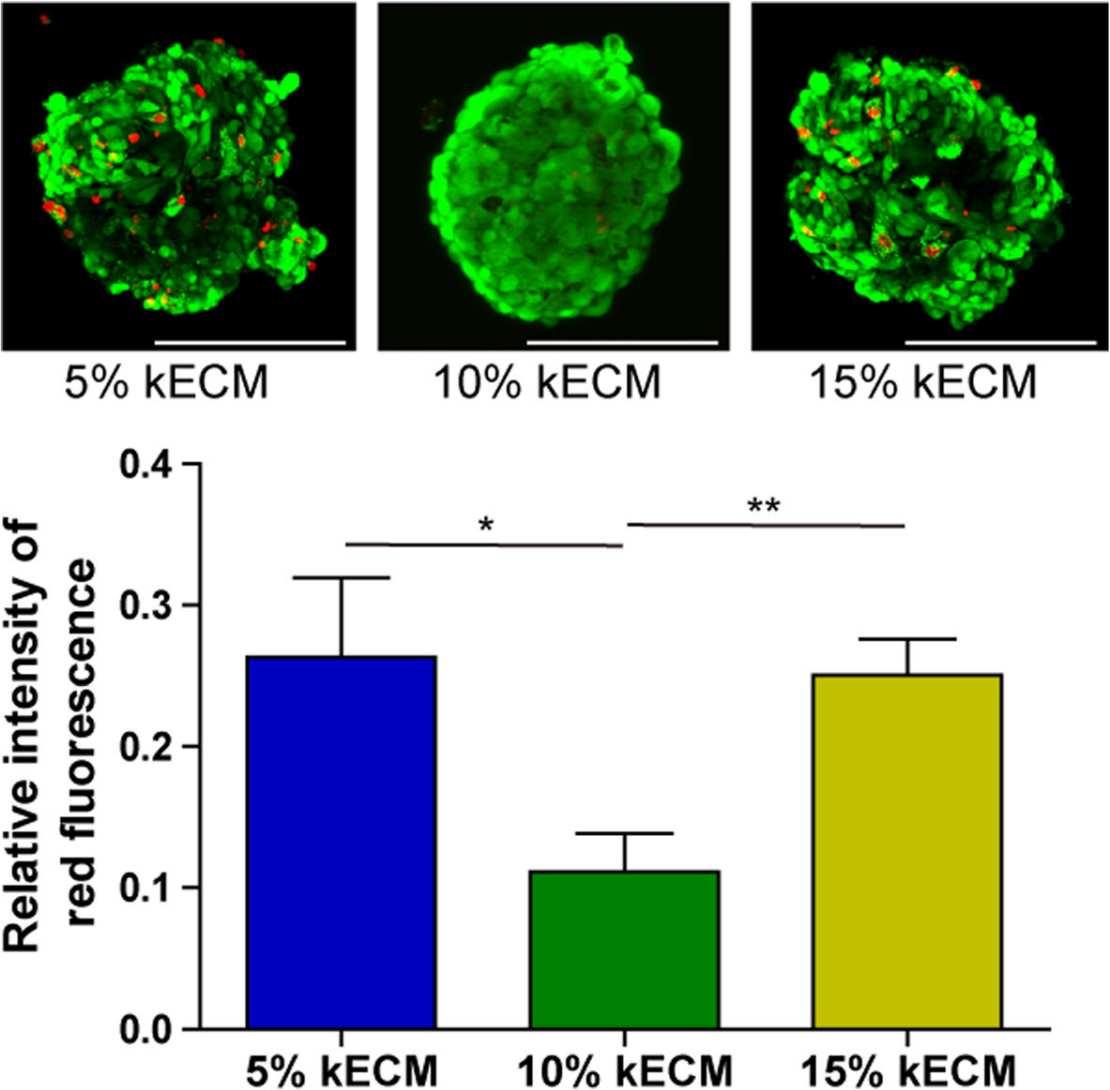
kECM concentration optimization via viability assay. Live/Dead staining was employed to evaluate the viability of USC-organoids in different kECM concentration. Green fluorescence indicated live cells, and red fluorescence-labeled dead cells. The red fluorescence intensity was quantified. With low kECM concentration, the 3D cell culture simulation effect was poor, and the ability to obtain renal cell phenotype was also poor. Otherwise, high kECM concentration would affect the viscosity of the medium and finally influence nutrition penetration. From the figure, the 10% kECM was the optimal concentration for organoid formation (scale bar 200 μm)

Live/Dead staining was employed to evaluate organoid viability. According to the above data in cell density optimization, USCs were seeded at the density of 5000 cells/well to generate 3D organoids. The KCM was supplemented with 5%, 10%, or 15% solubilized kECM. At day 14, organoids were harvested for Live/Dead staining. The results showed that the organoid cultured with 10% kECM were well self-organized and had the most green fluorescence (living cells) and least red fluorescence (dead cells), indicating the best viability, seen from Fig. 3. These findings revealed that 10% kECM was the optimal concentration for organoid formation.

The above data indicated the optimal cell seeding density was 5000 cells/well and optimal kECM additive concentration was 10% in our experimental system. As-formed USC-organoids were employed in the next assessments.

### Histology evaluation for 3D organoids

H.E. staining, immunofluorescence staining, and whole mount staining were applied for comparative observation for three groups of 3D organoids, USC-organoids, USC-organoids with kECM, and renal organoids. All organoids cultured for 14 days were submitted to the following staining assays.

**Fig. 4:**
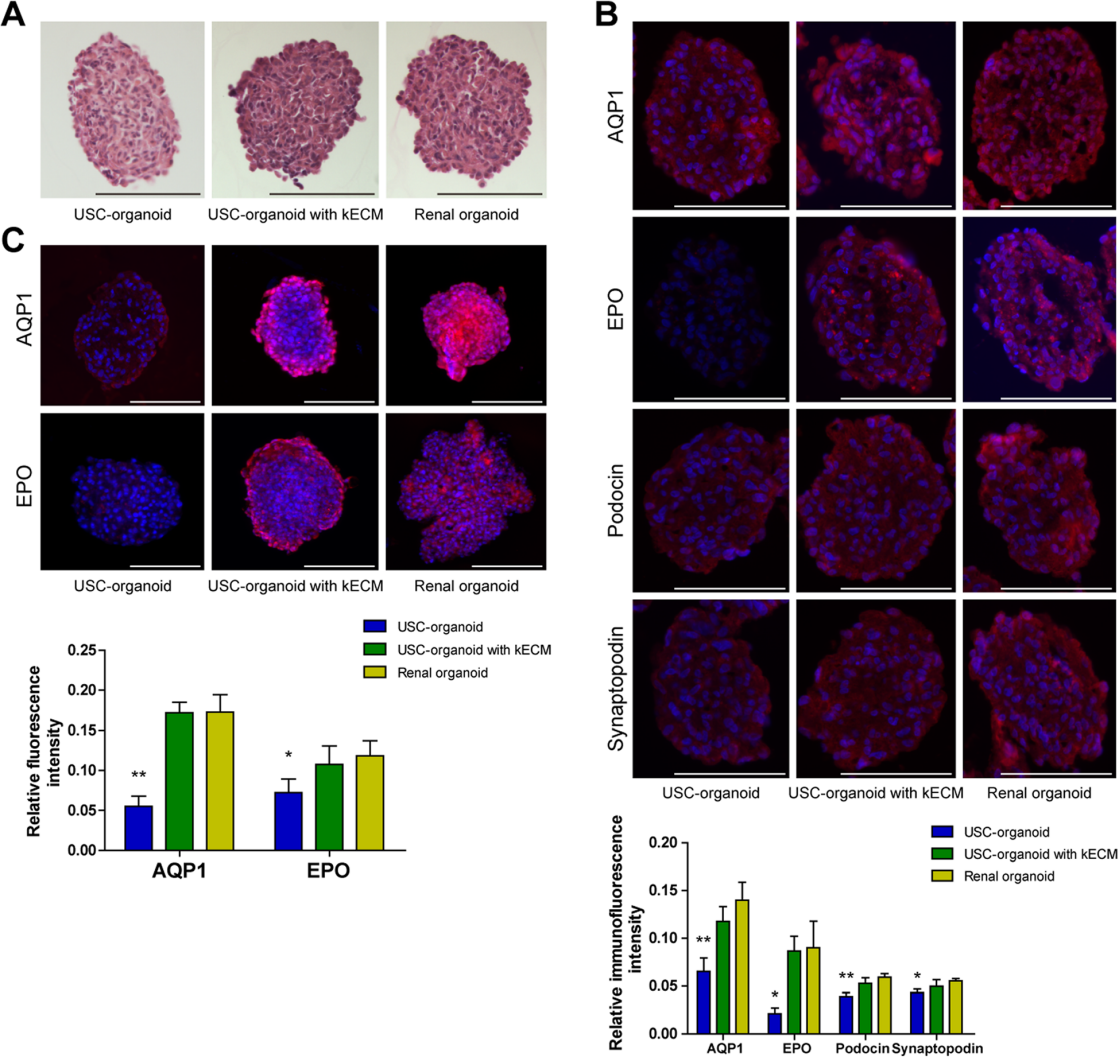
Histology of 3D organoids (USC-organoids, USC-organoids with kECM, and renal organoids). **a** H.E. staining. The cell nucleus was blue stained, and cytoplasm was red stained. **b** Immunofluorescence staining for specific proximal tubule AQP1, kidney endocrine product EPO, and kidney glomerular markers Podocin and Synaptopodin. AQP1, EPO, and Podocin and Synaptopodin were labeled as red and DAPI as blue. The fluorescence intensity was quantified. **c** Whole mount staining for AQP1 and EPO. Red fluorescence indicated AQP1 and EPO and DAPI as blue. The whole mount staining provided 3D vision. The fluorescence intensity was quantified (scale bar 200 μm)

Approximately, three groups of organoids looked uniform in cellular aggregation and compact in structure organization in H.E. staining. In Fig. 4a, the cell nucleus was blue stained, cytoplasm was red stained, and there was no large necrosis and voids in the organoids. In terms of cytoplasm staining, USC-organoids with kECM was closer to renal organoids, with more cytoplasm and deeper red staining.

Additionally, marker proteins for specific proximal tubule AQP1, kidney endocrine product EPO, and kidney glomerular Podocin and Synaptopodin were marked as red on sectioned organoids by immunofluorescence staining while DAPI as blue. In Fig. 4b, we found that three groups of 3D organoids showed similar patterns in terms of AQP1, indicating that they all possessed water metabolism function. The AQP1 dyeing intensity of USC-organoids with kECM was closer to renal organoids. Besides, USC-organoids with kECM had similar EPO expression level to renal organoids, but there was almost none EPO fluorescence in USC-organoids without kECM obviously. Furthermore, the Podocin and Synaptopodin dyeing intensity of USC-organoids with kECM was closer to renal organoids.

Moreover, the whole mount staining was performed to label the fluorescence of AQP1 and EPO in three-dimensional distribution. Figure 4c showed that USC-organoids with kECM were analogous to renal organoids to a large extent.

### Expression of renal-specific markers

**Fig. 5:**
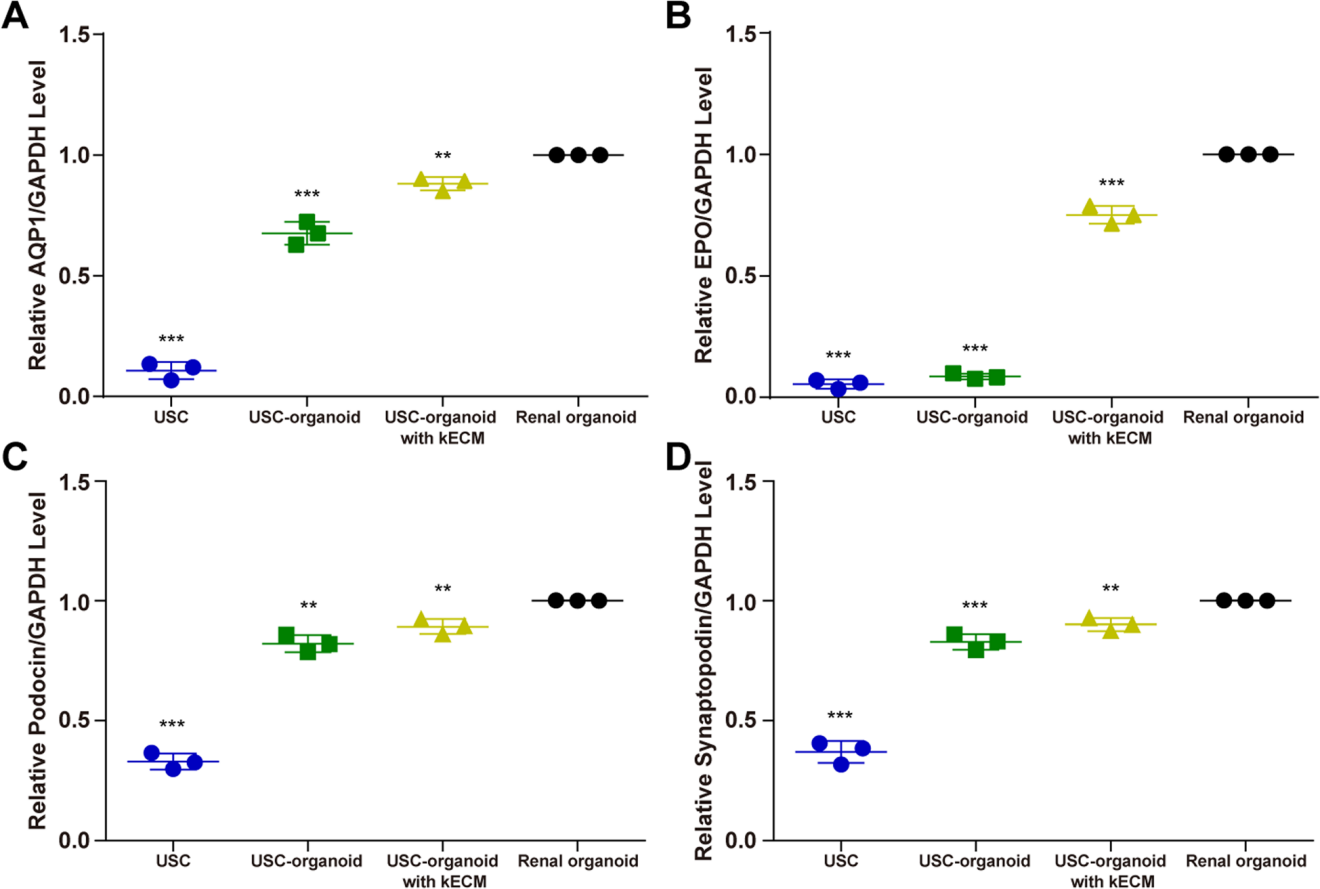
Expression of renal-specific markers in USCs and 3D organoids via RT-qPCR. **a** The expression level of specific proximal tubule AQP1. There was near none AQP1 expression in USCs, but three groups of organoids all had AQP1 expression. The AQP1 expression level of USC-organoids with kECM was between USC-organoids and renal organoids. **b** The expression level of kidney endocrine product EPO. Only USC-organoids with kECM and renal organoids had clear EPO expression. **c** The expression level of kidney glomerular marker Podocin. The Podocin expression level of USC-organoids with kECM was between USC-organoids and renal organoids. **d** The expression level of kidney glomerular marker Synaptopodin. The Synaptopodin expression level of USC-organoids with kECM was between USC-organoids and renal organoids. The AQP1, EPO, Podocin, and Synaptopodin expression level of USCs, USC-organoids, and USC-organoids with kECM were all significantly lower than renal organoids (three replicated samples for each group, data presented as mean ± SD)

RT-qPCR was performed to evaluate the above markers quantitatively at RNA level, and the relative level of gene expression is shown in Fig. 5. In Fig. 5a, compared to near none expression in USCs, three groups of organoids all had AQP1 expression, and the AQP1 expression level of USC-organoids with kECM was between USC-organoids and renal organoids (USC 0.107 ± 0.036, USC-organoid 0.675 ± 0.048, USC-organoid with 10% kECM 0.882 ± 0.027). In Fig. 5b, only USC-organoids with kECM and renal organoids showed clear EPO expression (USC 0.055 ± 0.019, USC-organoid 0.409 ± 0.035, USC-organoid with 10% kECM 0.751 ± 0.037). Figure 5c, d showed that Podocin and Synaptopodin expressions of USC-organoids with kECM were between USC-organoids and renal organoids (Podocin [USC 0.330 ± 0.034, USC-organoid 0.822 ± 0.036, USC-organoid with 10% kECM 0.894 ± 0.031]; Synaptopodin [USC 0.369 ± 0.047, USC-organoid 0.830 ± 0.033, USC-organoid with 10% kECM 0.901 ± 0.027]). The AQP1, EPO, Podocin, and Synaptopodin expression levels of USCs, USC-organoids, and USC-organoids with kECM were all significantly lower than renal organoids.

### Evaluation of USC-organoids as a nephrotoxicity screening model

**Fig. 6:**
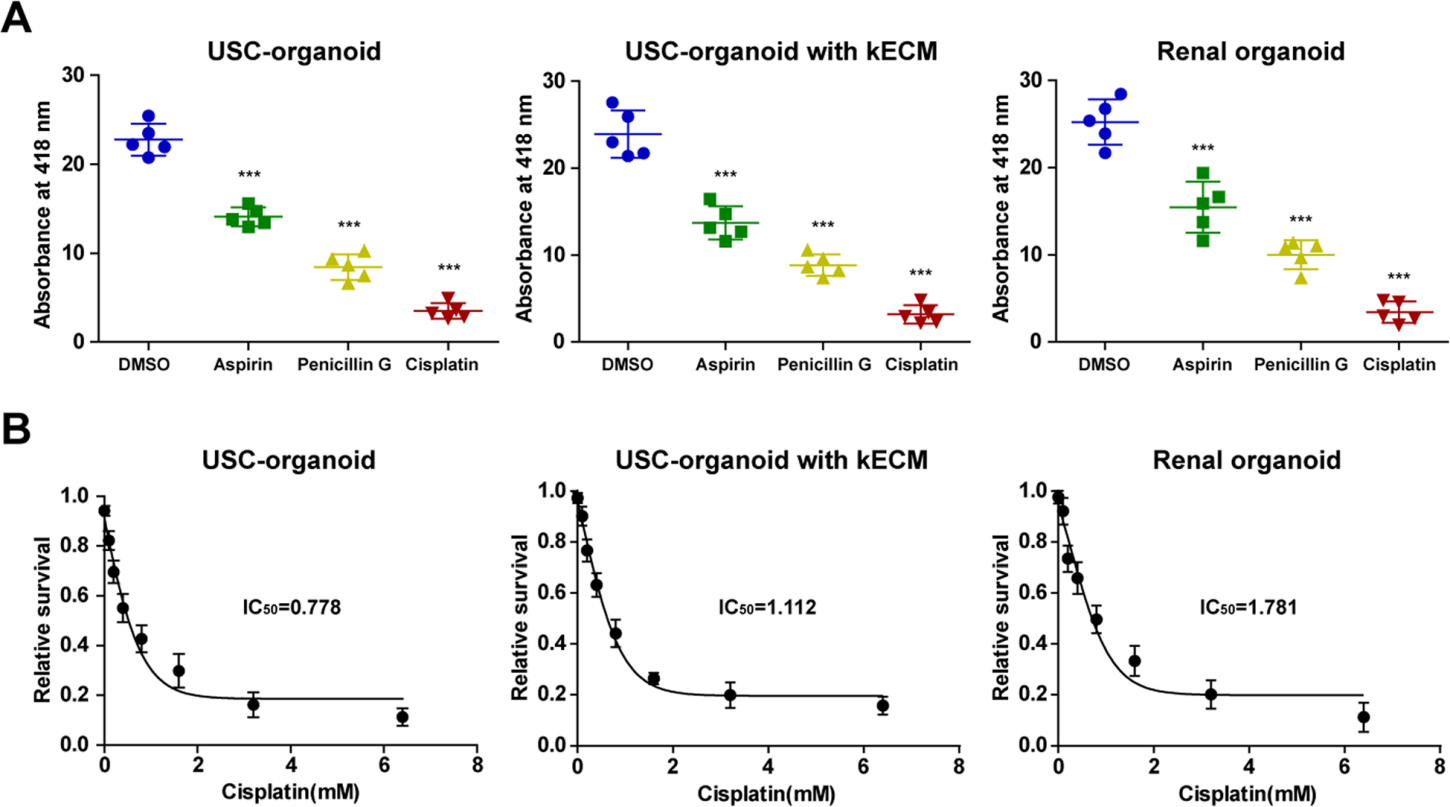
Application of USC-organoids in renal function and drug screening. **a** GGT activity measurement for renal function and drug screening in 3D organoids. Percentage decrease in the absorbance: USC-organoids (aspirin 38.2%, penicillin G 63.0%, cisplatin 84.6%), USC-organoids with kECM (aspirin 42.8%, penicillin G 63.1%, cisplatin 86.8%), renal organoid (aspirin 38.8%, penicillin G 60.4%, cisplatin 86.4%) (five replicated samples for each group, data presented as mean ± SD). **b** Dose-response curves of cisplatin in in 3D organoids. The IC_50_ of cisplatin in USC-organoids, USC-organoids with kECM, and renal organoids were 0.778 mM, 1.112 mM, and 1.781 mM, respectively (three replicated samples for each group, data presented as mean ± SD)

Drug toxicity test was carried out to assess the potential role of USC-organoids for nephrotoxicity screening. Similar trends were observed between USC-organoids with kECM and renal organoids in Fig. 6a. Significantly, the decreased absorbance at 418 nm was observed in aspirin, penicillin G, and cisplatin group than the control group, demonstrating that these drugs at normal drug concentration could exert similar drug-induced toxicity and the USC-organoids with kECM had similar drug screening ability to renal organoids.

The “relative cell survival” values and drug concentrations were fitted to dose-response curves to estimate the IC_50_ values of cisplatin. Respectively, the IC_50_ of cisplatin in USC-organoids, USC-organoids with kECM, and renal organoids were 0.778 mM, 1.112 mM, and 1.781 mM, marked in Fig. 6b.

## Discussion

This study successfully sketched the optimized process of USC-organoid generation with kECM and proved that USC-organoids had similar morphology, histology, gene expression, and nephrotoxicity screening ability to renal organoids. Firstly, the USCs were collected, and porcine-derived kECM was prepared to imitate the in vivo environment. Next, the optimal cell density and kECM concentration were determined by examining organoids with proliferative ability and viability. Finally, we evaluated the characteristics of USC-organoids and compared it with renal organoids by multiple assays. Briefly, our study developed a novel type of organoid, namely USC-organoids, and validated its identity and potential in nephrotoxicity screening to take the place of renal organoids or renal tissue models.

The functional renal cells are in great need in both clinic and laboratory, which have insufficient and unstable source, even worse the renal organoids for research. The urine-derived stem cells have many advantages compared to adult somatic stem cells and hPSCs. They can be collected in a noninvasive manner from an easily accessible source [41]. In addition, there are less ethical restrictions about urine cells [26]. On one hand, there were sparse studies implying the involvement of USCs in organoid generation. Usui et al. elucidated that prostate cancer organoids were successfully generated using dog urine cancer stem cells, and these organoids might become a useful tool for the research of cancer pathogenesis and treatment [42]. Besides, Wan et al. illustrated that differentiated urothelium was generated from stem cells isolated from the urine and the urothelium was like native urothelium phenotypically and functionally [28]. On the other hand, there were some studies concerning about urine-derived human-induced pluripotent (U-hiPSCs). Li et al. reported the success of retinal organoids differentiated from U-hiPSCs and revealed that they could be used in disease modeling, drug screening, personalized medicine, etc. [43]. Furthermore, Jiang et al. demonstrated that U-hiPSCs could be differentiated into cardiomyocytes in vitro and in vivo [41]. Moreover, Yi et al. elaborated that they differentiated U-hiPSCs into motor neurons, and U-hiPSCs were promising source for motor neuron disease modeling [29].

Here, we systematically generated USC-organoids with the help of kECM and verified its renal function preliminarily. Currently, several categories of ECM including animal-derived ECM (general or tissue-specific) and synthetic matrices have been developed for 3D culture [44]. Frans et al. indicated that animal tissue-derived ECM could improve the expansion, differentiation, and passaging of renal organoids [30]. They also reviewed that synthetic matrices might not only contribute to renal organoid culture but also enable the clinical grade renal organoids’ formation for cell therapy [30]. So far, animal tissue-derived ECM matrix is the only matrix reported to be used for organoid culture [30]. Further studies are needed to verify the kECM contribution to renal function of USC-organoids.

Several types of function can be assessed to identify renal characteristics including tissue-specific barrier and transport functions [30]. Takasato et al. demonstrated that renal organoid cells with a proximal tubular phenotype had the cubilin-mediated endocytosis function [19]. Freedman et al. elaborated that methotrexate and dextran could be transported into the lumen of renal organoids, and this was mediated by endocytosis [22]. In this study, we used EPO test to validate the renal function. EPO is an important glycoprotein hormone produced from renal fibroblasts, and it can stimulate red cell production [45, 46]. We proved that USC-organoids cultured with kECM had similar EPO expression pattern with renal organoids, indicating the endocrine function of USC-organoids.

Newly developed drugs including antibiotics and immunosuppressants usually have potential in nephrotoxicity, and it is critical to predicting drug nephrotoxicity before they are used clinically [3, 4]. Thus, novel and accurate models for screening drug nephrotoxic activity are urgently needed [5]. To date, isolated human primary renal cells and immortalized cell lines had been utilized for drug screening, but they showed low specificity, reproducibility, and accuracy [5]. Besides, ESCs and hiPSCs are unable to simulate distinct renal cells, kECM, vascularization, etc. [47]. Furthermore, rodent models are poor predictors of nephrotoxicity because of the species differences in gene expression [5]. Presently, renal organoids containing several types of renal cells are thought to be an effective approach for predictive toxicity [47]. So far, many studies reported that kidney organoids from hPSCs could be applied in drug toxicity testing [3, 48–50]. In our study, we demonstrated that USC-organoid was a promising drug nephrotoxicity screening tool. Aspirin and penicillin G are excreted by the kidneys and can exert an adverse effect on renal function [51]. Cisplatin may cause acute proximal tubular necrosis [52]. GGT is expressed in proximal tubule, and it is essential for the γ-glutamyl cycle, which plays a critical role in the detoxification of xenobiotics [18]. Its activity can be used to represent the renal cell function [18]. The GGT assay showed that renal function of USC-organoids and renal organoids both were impaired by aspirin, penicillin G, and cisplatin in a dose-dependent way.

## Conclusions

This study develops USC-organoids successfully and uses kECM to guide their renal function. We verify USC-organoid properties including morphology, histology, gene expression, and their potential application value for drug screening.

## Supplementary information

**Additional file 1.** The preparation process of kECM.

## Data Availability

Not applicable.

## Abbreviations

3D: Three-dimensional
USCs: Urine-derived stem cells
kECM: Kidney-specific extracellular matrix
H.E.: Hematoxylin and eosin
USC-organoids: USCs-derived organoids
AQP1: Aquaporin-1
EPO: Erythropoietin
hPSCs: Human pluripotent stem cells
hESCs: Human embryonic stem cells
hiPSCs: Human-induced pluripotent stem cells
EFM: Embryo fibroblast medium
KSFM: Keratinocyte serum-free medium
FBS: Fetal bovine serum
p0: Passage 0
HKCs: Human kidney cells
KCM: Kidney culture medium
DMEM: Dulbecco’s modified Eagle’s medium
GGT: Glutamyltransferase
IC_50_: Half maximal inhibitory concentration
SD: Standard deviation
RT: Room temperature
U-hiPSCs: Urine-derived human-induced pluripotent
