# Supplementary material for: Formation and optimization of three-dimensional organoids generated from urine-derived stem cells for renal function in vitro
Source: Stem Cell Res Ther. 2020 Jul 22;11:309. doi: 10.1186/s13287-020-01822-4 (PMC7374873; doi:10.1186/s13287-020-01822-4)
Supplement: Supplementary file 1 — Additional file 1. The preparation process of kECM. [file 13287_2020_1822_MOESM1_ESM.docx]

**Supplementary Figure 1 - The preparation process of kECM.** The discarded porcine kidney was cut into blocks and flash frozen at -80°C after thorough pre-rinsing blood vessels. The frozen blocks were further sectioned into slices. Kidney slices were immersed in distilled water and shaken on a rotary shaker at 200 rpm for 3 days at 4°C (water changed triply daily). Then, the slices were treated with 2% Triton X-100 for 4 days followed by 2% TX-100 + 0.1% NH_4_OH for 24 hours (TX-100 changed twice daily). The decellularized tissues were rinsed in distilled water for 2 days to eliminate TX-100. Next, the obtained ECM were lyophilized for 48 hours and grounded into powder with a freezer mill. ECM powder mixed with pepsin by 1 g: 100 mg in weight and sterilized by gamma irradiation (1 Mrad). Further, following procedures were carried out under sterile condition. The mixture was incubated in 0.1 mol/L hydrochloric acid and transferred to a 50 ml conical tube for centrifugation at 3 000 rpm for 15 min. The pellet was discarded repeatedly until the supernatant was clear. To get rid of remaining particles, the resulting suspension was filtered through a 0.2 µm syringe. Finally, neutralized by 1 mol/L NaOH to pH 7.0, the kECM extracts were ready for use.
